# Vision AI-Based Gamified Cognitive Prosthesis for Executive Function: Feasibility and Usability Study

**DOI:** 10.2196/74157

**Published:** 2025-10-06

**Authors:** Co Yih Siow, Yao-Hua Yang, Cheng-Jui Tsai, Wan-Wan Yang, Chaur-Jong Hu, Jia-Ying Sung, Jowy Tani

**Affiliations:** 1 Department of Physical Medicine and Rehabilitation, Taipei Medical University Hospital, Taipei Medical University, Taipei, Taiwan; 2 Biomed Innovation Center, Wan Fang Hospital, Taipei Medical University, Taipei, Taiwan; 3 School of Medicine, College of Medicine, Taipei Medical University, Taipei, Taiwan; 4 Department of Neurology, School of Medicine, College of Medicine, Taipei Medical University, Taipei, Taiwan; 5 Department of Neurology, Shuang Ho Hospital, Taipei Medical University, New Taipei City, Taiwan; 6 Department of Neurology, Wan Fang Hospital, Taipei Medical University, No.111, Sec. 3, Xinglong Rd., Wenshan Dist., Taipei, 116, Taiwan, 886 -2-6940-7930 ext 6940

**Keywords:** artificial intelligence, audiovisual stimulation, cognitive impairment, cognitive prosthesis, digital therapeutics, executive function, mild dementia

## Abstract

**Background:**

Dementia is a progressive neurodegenerative condition marked by cognitive decline and loss of functional independence. Among cognitive domains, executive dysfunction is a critical early contributor to reduced self-care capacity and increased caregiver burden. While cognitive assistive technologies have focused primarily on memory, few tools address executive function in real-time, daily tasks. To fill this gap, we developed a novel gamified cognitive prosthesis that integrates artificial intelligence (AI) and computer vision to guide users step-by-step through a simulated egg-cooking task. This system provides real-time audiovisual feedback to support planning, sequencing, and error correction.

**Objective:**

This study aimed to evaluate whether the AI-based cognitive prosthesis improves task completion time and executive function performance in individuals with mild dementia.

**Methods:**

We conducted a pilot study involving 12 patients with mild dementia and 7 age-matched healthy controls. Participants were asked to complete a 6-step gamified egg boiling task under 2 conditions: with and without guidance. The task was evaluated using a custom “Daily Task Completion Test” and a modified executive function performance test (EFPT) adapted to the cooking activity. Demographic and clinical data (age, sex, education, Mini-Mental State Examination, Clinical Dementia Rating, activities of daily living, instrumental activities of daily living, and Dementia Severity Rating Scale) were recorded. The System Usability Scale (SUS) was also collected postintervention.

**Results:**

In the mild dementia group, AI assistance significantly reduced median task completion time from 134.75 (IQR 92.50‐134.75) to 92.00 (IQR 65.00‐92.00; *P*=.03) seconds, and significantly improved the Executive Function Performance Test (EFPT) scores from 4.25 (IQR 1.75‐4.25) to 1.00 (IQR 0.00‐1.00; *P*=.005), reflecting a 31.7% improvement in efficiency and a 76.5% reduction in required assistance. No significant changes were observed in the control group. The mean SUS score was 80.53 (SD 24.97), indicating high usability. The AI system achieved a cumulative recognition precision of 0.93 (SD 0.07) and cumulative recall of 0.94 (SD 0.11).

**Conclusions:**

This pilot study provides preliminary evidence that an AI-based cognitive prosthesis can enhance executive function and task performance in individuals with mild dementia. The results support the feasibility of using real-time AI guidance in everyday tasks to promote independence. Given its modular design and promising usability profile, this system may serve as the foundation for future digital therapeutics targeting executive dysfunction. Larger, longitudinal studies are warranted to evaluate sustained cognitive and functional benefits.

## Introduction

The rising incidence and prevalence of dementia have led to a substantial global care burden [1,2]. Dementia represents a significant challenge as it gradually diminishes the self-sufficiency of individuals in their everyday routines and results in a notable deterioration in their overall well-being [3]. Consequently, this imposes a substantial burden on both the affected families and society [4-7]. Common causes of dementia include Alzheimer disease, vascular dementia, and frontotemporal dementia, with Alzheimer disease contributing to the largest part of the dementia population [8]. The caregiver burden of patients with Alzheimer disease was reportedly significantly higher compared to the burden borne by caregivers of older adults with psychosis, with spouses bearing the highest burden score [7]. The estimated number of people with dementia exceeded 50 million in 2019, and the cases will surpass 150 million in 2050 globally. Globally, the annual spending on individuals with dementia is over a trillion US dollars, while the forecasted cost for dementia will reach US $1.6 trillion in 2050 [8-10]. The societal burden of dementia is projected to reach epidemic levels across countries in the Asia-Pacific region [11]. Taiwan, similarly, is currently experiencing an increasing burden of dementia. Such statistics underscore the critical need for innovative interventions.

Over the past decade, various assistive technologies have been developed for individuals with dementia, with variable levels of success [12,13]. Memory has been a significant focus for the development of cognitive assistive devices, and previous studies have shown that cognitive prosthesis could be beneficial for patients with impaired facial memory [13,14]. The term cognitive prosthesis refers to a compensatory strategy to modulate the environment for rehabilitation through computer technology focusing on the individual’s functional activities [15]; in this study, the term refers to a device that augments the cognitive function of the user. Digital interventions for dementia have also been attempted in previous studies [16-20]. In terms of interventions for Alzheimer disease and the general mild-to-moderate stage of dementia, cognitive stimulation therapy and psychosocial interventions have reportedly been shown to preserve cognitive abilities longer than pharmacological treatments [8]. Cognitive rehabilitation, personalized to each individual’s needs, has also been reported to improve the activities of daily living (ADL) score and their cognitive functioning, providing evidence that cognitive assessments through various means, including cognitive assistive technology, may be useful as an intervention for all-cause dementia [21].

However, the global burden of various diseases’ impacts is receiving increasing attention based on the results of the global burden of disease study, which the World Health Organization supports [22]. There is a paradigm shift in dementia management toward promoting functional independence, a key factor directly influencing the quality of life and care burden. The role of executive function in dementia care has been increasingly recognized, and artificial intelligence (AI)-based tools are considered valuable for promoting functional independence [23].

Executive function is a multifaceted neuropsychological construct that can be defined as forming, maintaining, and shifting mental sets. It includes basic cognitive processes such as attentional control, inhibition, working memory, cognitive flexibility, judgment, and sequencing. The executive function enables people to formulate goals successfully, plan how to achieve them, and carry out the plans effectively, making it essential for daily life independence [24,25]. It plays a crucial role in everyday functioning and is indispensable for instrumental activities of daily living (IADL) [25]. Numerous studies have pointed out that improvements in IADL and Executive Function Performance Test (EFPT) scores were associated with improved quality of life [26,27], reduced incidents of home accidents [28], decreased caregiver burden [29], and lower cost of care [30]. This highlights the importance of shifting focus to executive functions in dementia assessment and treatment.

Although executive function plays a vital role in daily functioning, few assistive technologies have been developed to provide real-time assistance for executive functioning, helping patients complete daily tasks [31]. There has been computerized cognitive training with the NeuroNation MED app in which the users have shown significant improvement in global cognition in mild cognitive impairment (MCI) patients compared to controls [32]. Another randomized clinical trial featuring Constant Therapy app in over 24 weeks for Alzheimer’s patients also showed high adherence and task improvements [33]. Virtual reality and gaming-based therapies also have encouraging cognitive benefits, with moderate-to-large gains in global cognition, memory, and executive skills in MCI and dementia patients [34]. Virtual reality–based shopping programs have also been developed to screen for MCI or as therapy, with promising results for both intended uses [35,36]. However, there have been few assistive technologies using AI to improve executive functions.

In response to this gap, a novel AI-based cognitive prosthesis was introduced, integrating gamification and sensor technology to specifically aid individuals with dementia in the training of their executive functions. The prosthesis simulates everyday tasks—cooking egg using interactive tools—designed to replicate the sequential planning, decision making, and motor coordination required in daily life. The present study investigated the effectiveness of an AI-based cognitive prosthesis on individuals with dementia to improve executive functions.

## Methods

### Study Settings and Cohort

**Figure 1.**
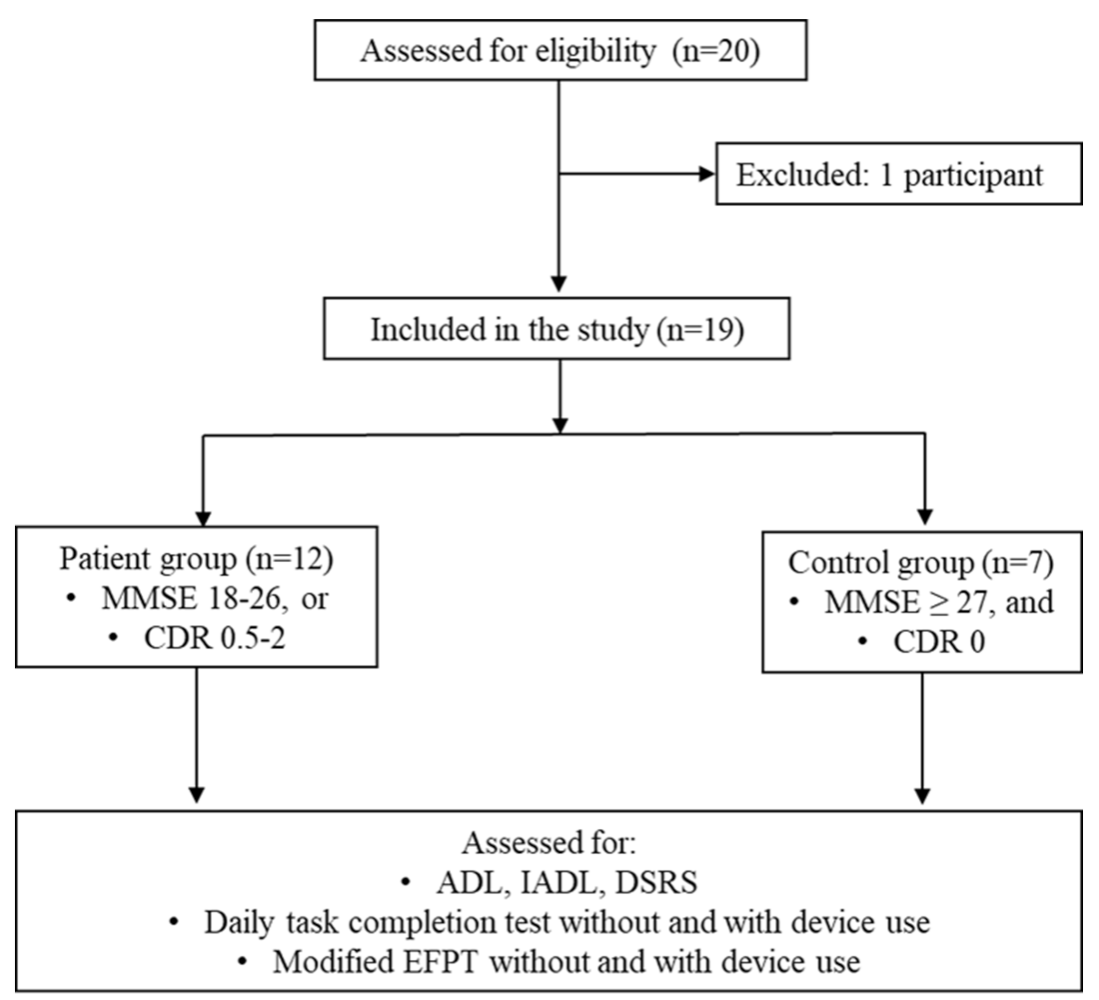
Flow chart depicting the experimental process. MMSE: Mini-Mental State Examination; CDR: Clinical Dementia Rating; ADL: activities of daily living; IADL: instrumental activities of daily living; DSRS: Dementia Severity Rating Scale; EFPT: Executive Function Performance Test.

Individuals with mild dementia (patient group) and healthy individuals (control group) were recruited from Wan Fang Hospital, Taipei Medical University. Performance differences between the patient and control groups were compared under 2 conditions: with or without the assistance of cognitive guidance devices. A flowchart outlining the experimental process is presented in Figure 1. Mini-Mental State Examination (MMSE) and Clinical Dementia Rating (CDR) tests were done as the participant selection criteria; the age, gender, and education level of the participants in both groups were also recorded. The participants included in the study were assessed for their ADL, IADL, and Dementia Severity Rating Scale (DSRS) at baseline before being asked to use the cognitive prosthesis. After completing the tasks, the participants were assessed with the modified EFPT and asked to fill out the System Usability Scale (SUS) questionnaire.

### Eligibility Criteria

In total, 12 individuals with mild dementia and 7 healthy individuals were recruited into the study. Individuals with mild dementia were eligible to be enrolled in the study if they met either of the following conditions: (1) MMSE score between 18‐26, or (2) CDR score between 0.5‐1.0 [37]. The age-matched healthy control participants’ eligibility criteria were as follows: (1) MMSE score equal to or greater than 27 [38] and (2) CDR score of 0.

Individuals with the following characteristics were excluded from the study: (1) inability to read and understand Chinese, (2) physical impairments, such as upper limb impairments, vision, and hearing impairments, which might hinder the subjects’ ability to undergo testing, (3) age less than 60 years, and (4) have not received more than six years of education. All participants were provided written and verbal explanations of the experiment and gave written informed consent.

### Ethical Considerations

All participants were recruited from Taipei Municipal Wan Fang Hospital, Taipei, Taiwan. The study procedures adhered to the Declaration of Helsinki. The study protocol was approved by the Joint Institution Review Board of Taipei Medical University (Protocol number: N202204004). All participants provided written informed consent after receiving both written and verbal explanations of the study. All participants’ identities remained anonymous and undisclosed for privacy and confidentiality protection. No compensation was offered to the participants.

### Intervention: Executive Function Cognitive Prosthesis for Simple Cooking

**Figure 2.**
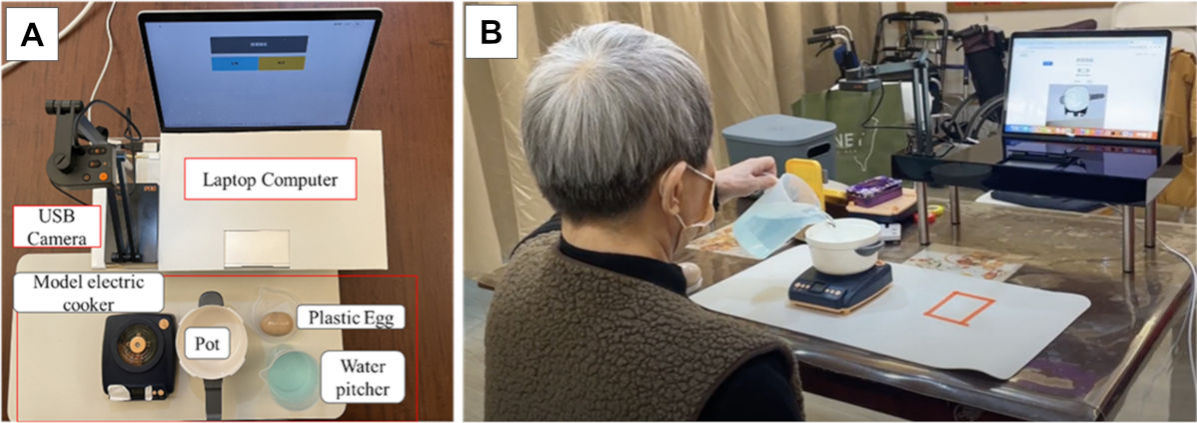
The gamified cognitive prosthesis setup. (**A**) Overall system for the simple cooking task. (**B**) A patient performing the task with the help of the cognitive prosthesis.

The novel cognitive prosthesis system consisted of a laptop, a universal serial bus camera connected to the laptop computer, and daily task objects. An AI-based executive function cognitive prosthesis software was installed on the laptop computer. Daily task objects—a model electric cooker, a plastic pot, a plastic egg, and a water pitcher—were used in the simulated egg-cooking task. The gamified cognitive prosthesis setup and experimental environment are depicted in Figure 2A and B.

In this study, the participants were instructed to perform a simple cooking task—boiling an egg—with or without visual and auditory cues provided by the executive function cognitive prosthesis.

The executive function cognitive prosthesis software was designed to assist users through the necessary steps of the egg boiling task. It analyzed visual data captured by the universal serial bus camera in real-time using a machine learning model to determine the state of each step of the task the participant has done. The machine learning model was trained using the YOLO v5 framework (Ultralytics), and the software was designed to operate on the Web platform. The software was configured to recognize the state of each task step by performing object recognition on the daily task objects—such as an electric cooker, pot, eggs, and water pitcher—by the machine learning model. The software would determine whether each task step is being performed correctly, record the time for each step completed, and then provide visual—both text and graphical—and audio instructions on the next step.

The cognitive prosthesis system incorporates 2 AI modules. The first module uses the Mediapipe hand detection model to monitor the participant’s actions, capturing key metrics such as initiation time, task duration, and task completion time [39]. The second module is a custom-trained model based on the YOLOv5 image recognition model [40], which identifies object locations and subsequently assesses task completion status. For custom training, we used 472 images for the training dataset, 93 images for the validation dataset, and 80 images for the test dataset, achieving an accuracy of 95.42%.

To develop the object detection model, we adopted transfer learning, initializing from pretrained weights on the COCO dataset, and fine-tuned the model using our custom dataset of 472 labeled images, covering approximately 20 distinct object classes. The sample images of the training dataset can be found in Figure S1 in Multimedia Appendix 1. Model training was conducted on Google Collab Pro using an NVIDIA Tesla T4 GPU with 16GB of VRAM. We adhered to the standard YOLOv5 training pipeline and employed a comprehensive toolset, including PyTorch for model architecture and training, OpenCV for image preprocessing and augmentation, and NumPy and Pandas for data manipulation. Visualization of model performance was carried out using Matplotlib and Seaborn. In addition, the Ultralytics command-line interface and YAML Ain’t Markup Language (YAML)–based configuration files facilitated customizable and reproducible hyperparameter tuning. This integrated setup enabled efficient experimentation while ensuring transparency and reproducibility in our workflow.

The model was trained using the default YOLOv5s input resolution (640×640), with a batch size of 16. Each training cycle comprised 250 epochs, a number selected to ensure model convergence given the relatively small dataset. Training was conducted on Google Colab Pro using a T4 GPU, with each cycle requiring approximately 100‐130 minutes (1.7‐2.2 h), depending on runtime conditions and GPU availability. Following each training cycle, we conducted a statistical evaluation that included calculation of mean average precision, class-wise precision, recall, and *F*_1_-scores, as well as confusion matrix generation. This analysis phase added an additional 3‐5 minutes per cycle. Consequently, each full training-evaluation iteration took approximately 105‐135 minutes. The process was repeated across 5‐7 iterations, with hyperparameters adjusted between runs to monitor performance trends and support generalizability of the model.

Camera data were collected every 5 ms and were processed using the aforementioned modules for inference. To ensure recognition stability, a rolling average over 5 frames was used. The recognition results and task progress were integrated continuously to update the system feedback.

### Cognitive Assistance and Clinical Assessments

All participants underwent neuropsychological tests and questionnaires, including ADL, IADL, and DSRS. The participants were then given approximately 2 minutes to read and familiarize themselves with the steps involved in the cooking task. The individuals with dementia were asked to perform the egg boiling task without any assistance from the cognitive prosthesis and assessed for a “daily task completion test” designed for the present study. Each of the 6 task steps was scored on the basis of task performance accuracy and completion time: 2 points for fully correct, 1 for partially incorrect, and 0 for incorrect performance. The perfect score was 12 points, indicating successful completion of all steps.

**Figure 3.**
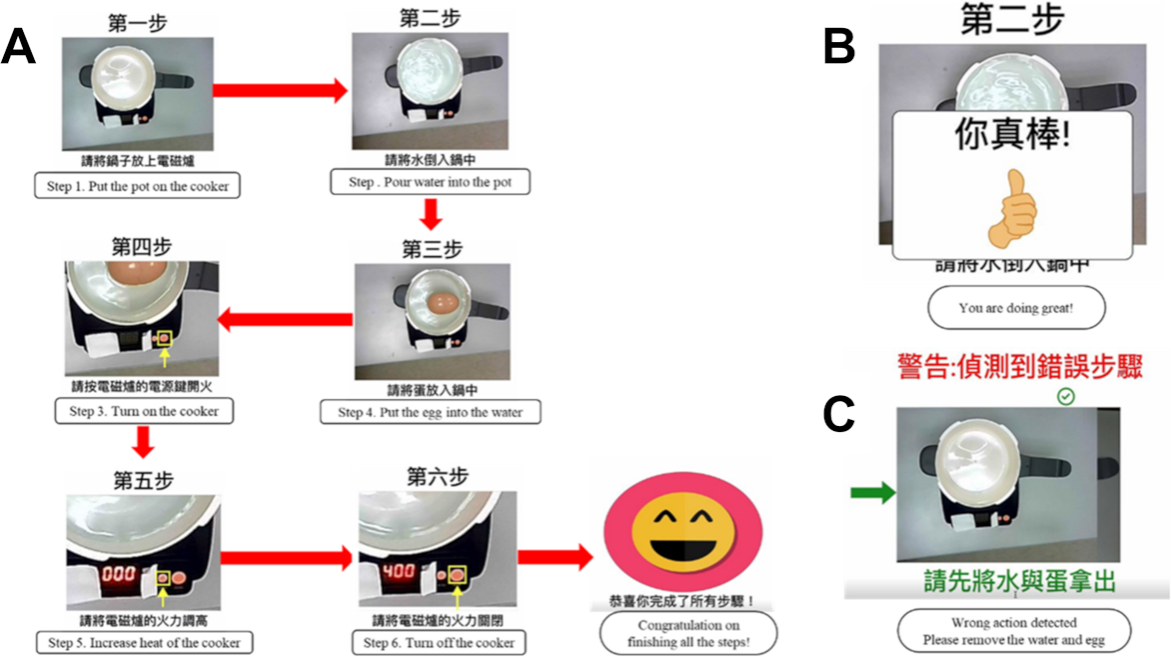
Egg-boiling task of the cognitive prosthesis overview. (**A**) Step-by-step egg cooking process with visual and auditory assistance of a cognitive prosthesis. (**B**) A screenshot showing the correct execution of the first step and transition to the second step, and (**C**) a screenshot showing a mistake in the second step and instructions for correction.

Subsequently, the participants repeated the egg boiling task with the assistance from the cognitive prosthesis device and were assessed for a “daily task completion test” again, as illustrated in Figure 3A. Guided by the system, the participants would proceed through the steps. Upon correct completion of each step, the system would provide positive reinforcement by displaying the message “great” (Figure 3B), after which the next step was prompted. If a step was performed incorrectly, the system would provide corrective instructions, requiring the participant to repeat the step correctly before proceeding (Figure 3C).

Executive function was evaluated using a modified EFPT that was adapted from the original EFPT of simple cooking task. The scoring ranged from 0 to 25, based on a 5-level cueing system: 0 (no cue required); 1 (verbal assistance); 2 (gestural assistance); 3 (direct verbal assistance); 4 (physical assistance); 5 (task performed by the assessor). A higher score indicates greater executive dysfunction.

Following the experimental task, participants or their caregivers completed the SUS questionnaire, designed to effectively distinguish user-friendly systems from less efficient ones on the basis of ease of use and efficiency.

### Object Recognition Algorithm Performance Evaluation

The computing time of the object recognition algorithm was evaluated across the 6 sequential task steps. For each step, the model’s processing time was measured and its cumulative precision and recall were evaluated.

### Statistical Analysis

The participant clinicodemographic characteristics, modified EFPT scores, task completion time, and completion score were summarized using descriptive statistics. Between-group differences were analyzed using the Mann-Whitney *U* test, whereas the sex distribution differences were analyzed using Fisher exact test. Performance differences before and after assistive device use for each participant were evaluated using the Wilcoxon signed-rank test. User experience data were analyzed descriptively. The classification model performance was assessed using a confusion matrix, with statistical significance set at *P*<.05. Processing delay times for each step of the executive function AI cognitive prosthesis were calculated and are presented as mean (SD) values. The diagram illustrating the statistical analysis done in the study can be found in Figure S2 in Multimedia Appendix 2.

## Results

### Cohort Characteristics

Clinicodemographic characteristics of the study participants are shown in Table 1. One patient was excluded from the study due to refusal to participate. There is no significant difference in age (*P*=.13) or gender (*P*=1.0) between the control group and the patient group after comparison. No significant differences were observed between the patients and control groups in terms of years of education (10, IQR 10-15 vs 12, IQR 12-16; *P*=.38). However, the patient group had significantly lower MMSE score (24.25, IQR 18.75‐24.25 vs. 30.00, IQR 30.00‐30.00; *P*<.001), DSRS scores (18.00, IQR 4.25‐18.00 vs 0.00, IQR 0.00‐0.00; *P*<.001), and IADL scores (*P*=.002), as well as significantly higher CDR score (*P*=.008). However, the ADL score difference between the control and patient group was not significant (*P*=.06) (Table 1).

**Table 1.** Demographic and clinical characteristics of the patient and control group.

Variables		Patient group (n=12)	Control group (n=7)	*P* value
Demographic profile				
	Age (years), median (IQR)	78.50 (75.25‐83.75)	72.00 (71.00‐72.00)	.13
	Sex-(male), n (%)	3 (25)	1 (15)	>.99
	Education (years), median (IQR)	15.00 (10.00‐15.00)	12.00 (12.00‐16.00)	.38
Clinical profile, median (IQR)				
	MMSE^a^	24.25 (18.75‐24.25)	30.00 (30.00‐30.00)	<.001
	CDR^b^	1.00 (0.50‐1.00)	0.00 (0.00‐0.00)	.008
	ADL^c^	100.00 (88.75‐100.00)	100.00 (100.00‐100.00)	.06
	IADL^d^	5.50 (3.00‐5.50)	8.00 (8.00‐8.00)	.002
	DSRS^e^	18.00 (4.25‐18.00)	0.00 (0.00‐1.00)	<.001

a MMSE: Mini-Mental State Examination.

b CDR: Clinical Dementia Rating.

c ADL: activities of daily living.

d IADL: instrumental activities of daily living.

e DSRS: Dementia Severity Rating Scale.

### Comparison of Task Completion Test in Both Groups

Table 2 shows the task performance results for the patient and control groups. At baseline, the patients have longer task completion time (seconds; 134.75, IQR 92.50‐134.75 vs 34.00, IQR 32.00‐42.00; *P*=.001) and a worse modified EFPT score (4.25, IQR 1.75‐4.25 vs 0.00, IQR 0.00‐0.00; *P*=.001) than the healthy controls.

**Table 2.** Comparison of task completion test for the advanced task module in both groups.

		Patient group (n=12)	Control group (n=7)	*P* value between groups
Task completion time (seconds)				
	Without device assistance, median (IQR)	134.75 (92.50‐134.75)	34.00 (32.00‐42.00)	.001
	With device assistance, median (IQR)	92.00 (65.00‐92.00)	39.00 (32.50‐40.00)	.001
	*P* value without versus with device	.03	.91	—^a^
Task completion score				
	Without device assistance, median (IQR)	12.00 (10.00‐12.00)	12.00 (12.00‐12.00)	.008
	With device assistance, median (IQR)	12.00 (10.75‐12.00)	12.00 (12.00‐12.00)	.06
	*P* value without versus with device	.14	>.99	—
Modified EFPT^b^ score				
	Without device assistance, median (IQR)	4.25 (1.75‐4.25)	0.00 (0.00‐0.00)	.001
	With device assistance, median (IQR)	1.00 (0.00‐1.00)	0.00 (0.00‐0.00)	.10
	*P* value without versus with device	.005	>.99	—

a Not applicable.

b EFPT: executive function performance test.

Although the use of assistive device led to no significant improvement in the task completion score, the patient group exhibited significant improvements in the time taken and modified EFPT score after using assistive devices. In the patient group, the time (seconds) required to complete the task significantly (*P*=.03) decreased from 134.75 (IQR 92.50‐134.75) to 92.00 (IQR 65.00‐92.00) after the device was used. Also, their modified EFPT score significantly (*P*=.005) decreased from 4.25 (1.75‐4.25) points before using assistive devices to 1.00 (IQR 0.00‐1.00) points with use. In contrast, there were no significant differences in the performance of the control group, whether they used assistive devices or not (Table 2).

### SUS

The SUS for executive function AI cognitive prosthesis device prototype for both groups is shown in Table 3. The SUS score interpretation is as follows: significant usability issues for <50 score, marginal usability for ≥50 to <68, acceptable usability for ≥68 to 80, and excellent usability for score above 80.

The overall SUS score obtained in this study was 80.53 (SD 24.97); a grade A categorization may be given, denoting acceptable usability. The overall level of the SUS score shows superior performance.

**Table 3.** The mean computing time for task completion steps.

	Computing time (ms), mean (SD)	Cumulative precision, mean (SD)	Cumulative recall, mean (SD)
Total Steps	11,720 (2.27)	0.93 (0.07)	0.94 (0.11)
Step 0	1062 (0.44)		
Step 1	1462 (0.99)		
Step 2	3125 (0.89)		
Step 3	1125 (0.81)		
Step 4	1625 (0.81)		
Step 5	2091 (0.83)		
Step 6	1231 (0.73)		

### Device Object Recognition Machine Learning Algorithm Performance of the Advanced Task Module

The cumulative precision of the object recognition machine learning model was 0.93 (SD 0.07), while the cumulative recall was 0.94 (SD 0.11). Table 3 shows the mean computing time for task completion steps, with the mean computing time for all steps being 11,720 (SD 2.27 ms; Table 3).

## Discussion

### Insight From the Cognitive Prosthesis Study

We evaluated the efficacy of an executive function cognitive prosthesis in aiding individuals with mild dementia. The device usage led to 76.5% improvement in the modified EFPT scores and 31.7% reduction in task completion time among patients with mild dementia, indicating improvement in executive function-related tasks. These findings suggest that the cognitive prosthesis has the potential to improve the patients’ daily activities.

### Differences in Baseline Profile of Patient Group

At baseline, the patients had significantly lower MMSE, IADL, and DSRS scores, as well as higher CDR, compared to the healthy control group; this confirmed the cognitive impairments in the patient group. During the simple cooking task completion, the patients required significantly longer time to complete the steps, along with lower task completion score and worse modified EFPT score; these reflected the deficit in daily task executive abilities in the patient group. These outcomes underscore the need for assistance among dementia patients in performing tasks.

### Effects of Cognitive Prosthesis Use on the Patients and Healthy Controls

To address the diverse training requirements of each subject and the different objects familiar to elderly individuals in different regions, we designed this system with a particularly scalable nature from the outset. Each time a new assistive module is designed, the process involves: (1) Designing the task workflow, (2) Collecting correct and incorrect workflow steps as training data for the image recognition model, and (3) Configuring the configuration file to adjust commands, recognition logic, and scoring methods. With a dedicated team of 3 (a designer, an AI engineer, and a system engineer), it takes approximately 2‐4 weeks to complete, demonstrating significant scalability.

The egg boiling task was selected as the initial AI daily task module for patients with mild dementia to assist with executive function. Usage of the cognitive prosthesis device enabled the patient group to complete tasks more efficiently. Notably, the modified EFPT scores showed significant improvement with the use of the device, suggesting that the cognitive prosthesis can enhance independence in performing simple daily tasks.

### SUS and Device Performance for the Cognitive Prosthesis

The adequate overall SUS score obtained, together with the excellent machine learning performance, did demonstrate the usability of the device.

### Toward the Cognitive Digital Therapeutic System

As the cognitive prosthesis system showed potential to improve EFPT during device use, this implicates the potential of using the similar technology to develop a digital therapeutics system (DTx). DTx should be able to assist the patients to have a longstanding improvement in executive function following regular training. The pilot study results of the integrated task DTx for healthy participants showed improved task completion scores and EFPT. Future interventional study is needed to ascertain whether the effects of cognitive prosthesis on individuals with dementia are sustained, in terms of long-term improvements in IADL and EFPT after extended use.

### Limitations

However, this study encountered some difficulties during its execution, and certain limitations should be considered. First and foremost, it is crucial to recognize that this research represents a single experimental intervention for the patients. As a result, it is uncertain whether the observed enhancements in the patients’ executive functions will persist or further develop in subsequent evaluations. Second, the number of participants fell short of our initial expectations. Even though the task completion scores of the patient group showed no significant change before and after device assistance, probably due to the limited sample size, the task completion time still significantly decreased by 31.7%.

### Future Work and Recommendation

Future studies with extended intervention periods are warranted to obtain more robust results and a thorough grasp of the long-term influence of cognitive prosthesis technology on executive function. Building on the results of this study, future research will expand to larger sample sizes to gather more substantial evidence. We aim to rapidly and efficiently develop AI-based cognitive prosthesis tailored to individual training needs, including hand operations, cognitive processes, and other areas. These developments will consider the cultural backgrounds of users to ensure the assistive technology meets diverse requirements. In the next research phase, we will focus on an integrated task module, specifically the shopping module, using the executive function cognitive prosthesis. We have high expectations for this phase and anticipate recruiting more participants to further demonstrate the efficacy of the executive function cognitive prosthesis.

### Conclusion

In conclusion, this study indicates that implementing cognitive prosthesis effectively improved executive function among individuals with mild dementia. The use of visual and auditory cues by the cognitive prosthesis reduced the task completion time in individuals with dementia. The cognitive prosthesis also showed high usability rating, indicating that it can be feasibly used by older adults. While further research is warranted to explore the potential of this intervention, it is expected that extending the use of cognitive prosthesis holds significant promise in dementia care and rehabilitation interventions. Maximizing their applicability is imperative to enhance the overall quality of care and support those people affected by cognitive impairment.

## Supplementary material

10.2196/74157 Multimedia Appendix 1 Sample images of training dataset.

10.2196/74157 Multimedia Appendix 2 Statistical analysis flow diagram.

## Abbreviations

ADL: activities of daily living
AI: artificial intelligence
CDR: Clinical Dementia Rating
DSRS: Dementia Severity Rating Scale
DTx: digital therapeutics system
EFPT: Executive Function Performance Test
IADL: instrumental activities of daily living
MCI: mild cognitive impairment
MMSE: Mini-Mental State Examination
SUS: System Usability Scale
