# Supplementary material for: Vision AI-Based Gamified Cognitive Prosthesis for Executive Function: Feasibility and Usability Study
Source: JMIR Serious Games. 2025 Oct 6;13:e74157. doi: 10.2196/74157 (PMC12500313; doi:10.2196/74157)
Supplement: Multimedia Appendix 1 [file games-v13-e74157-s001.docx]

Appendix 1. Sample images of the training dataset

**Figure S1. Sample images used in the training dataset with labelled objects.**


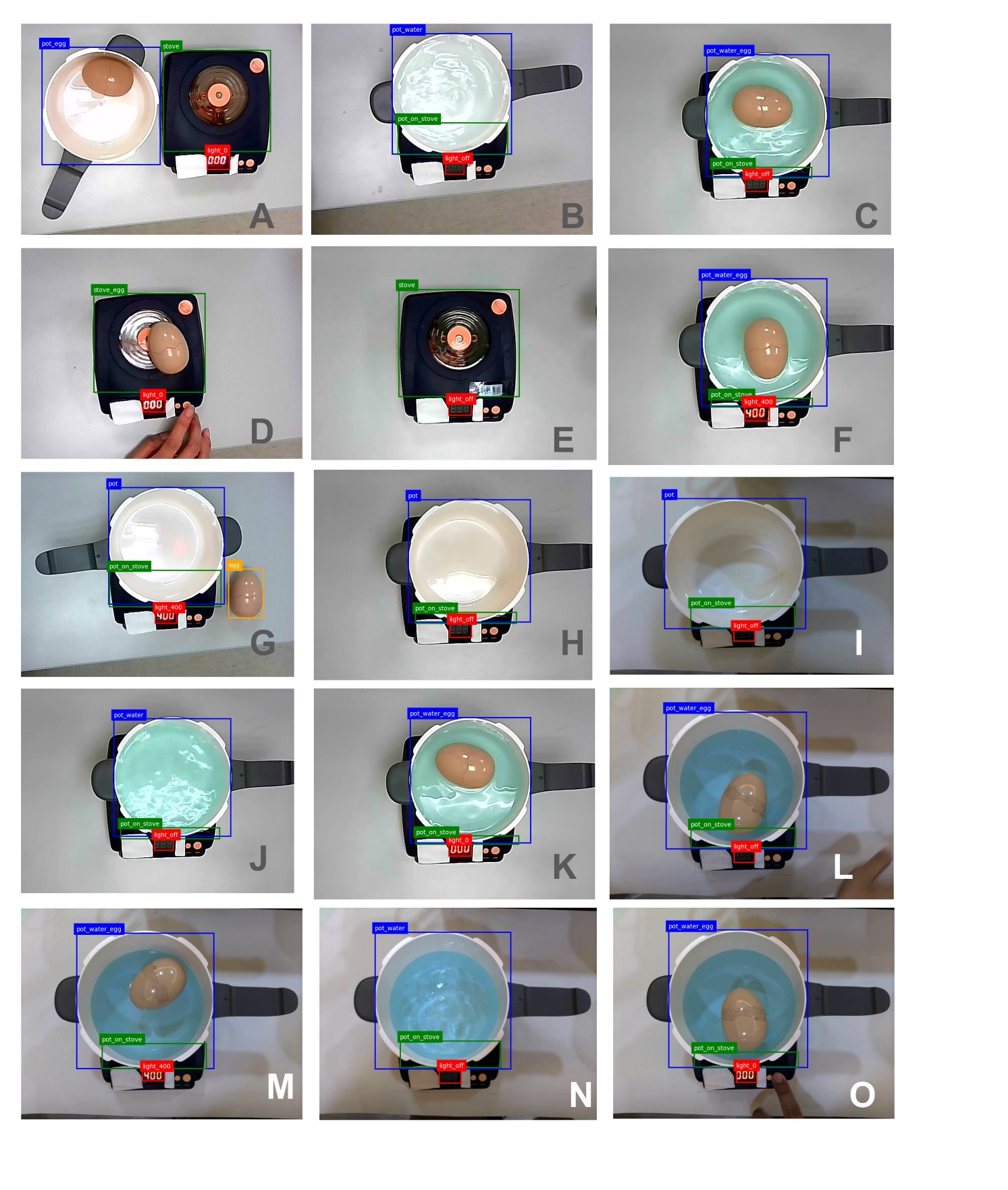


An egg in an empty pot beside the stove, stove is on (A); A pot filled with clear water placed on a turned-off stove (B); An egg in a water-filled pot, placed on a turned-off stove (C); An egg directly placed on the stove, stove is on at 0 (D); A turned off stove; An egg in a water-filled pot, placed on a turned-on stove at 400 setting (F); An empty pot on turned on stove at 400, egg is outside the pot (G); An empty pot on turned-off stove (H); An empty pot placed on a turned-off stove (I); A water-filled pot placed on a turned-off stove (J); An egg in a water-filled pot, placed on a turned-on stove at 0 setting (K); An egg in a water-filled pot, placed on a turned-off stove (L); An egg in a water-filled pot, placed on a turned-on stove at 400 setting (M); A water-filled pot on a turned off stove (N); An egg in water-filled pot, the pot is placed on a turned on stove at 0 setting (O).
