# Supplementary material for: Vision AI-Based Gamified Cognitive Prosthesis for Executive Function: Feasibility and Usability Study
Source: JMIR Serious Games. 2025 Oct 6;13:e74157. doi: 10.2196/74157 (PMC12500313; doi:10.2196/74157)

Appendix 2. Statistical analysis diagram and rationale

**Figure S2. The flow diagram of statistical analysis in the study.**

MMSE = mini-mental state examination; CDR = clinical dementia rating; ADL: activities of daily living; IADL: instrumental activities of daily living; SUS: System Usability Scale; EFPT: executive function performance test


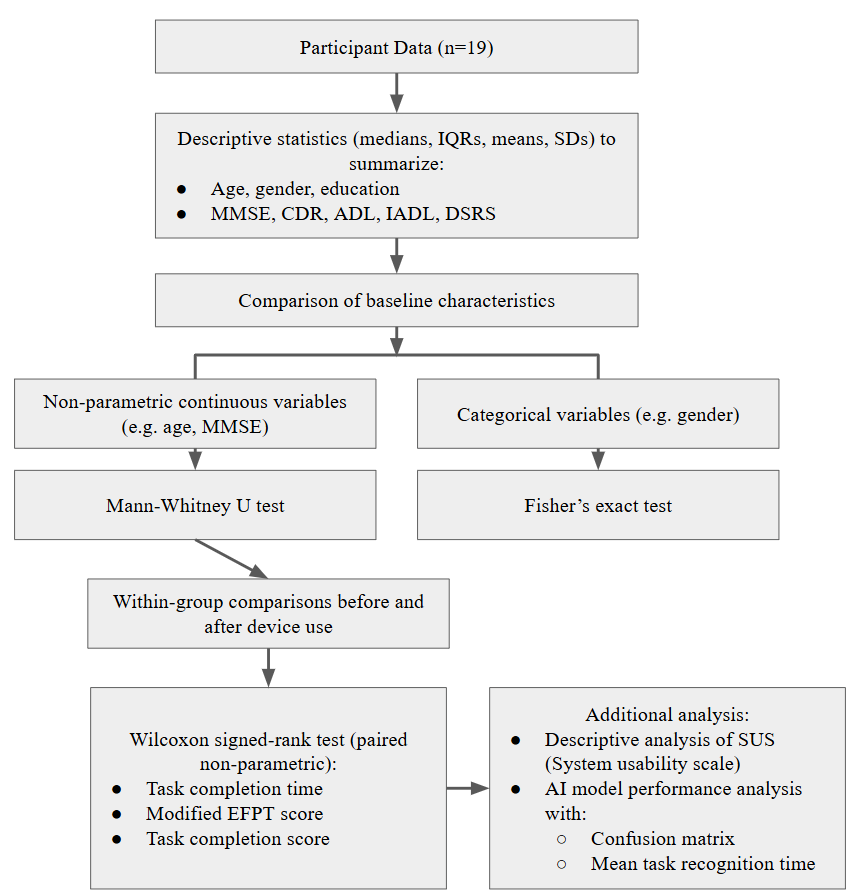

Supplement: Multimedia Appendix 2 [file games-v13-e74157-s002.docx]
